# Impact of Menthol Smoking on Nicotine Dependence for Diverse Racial/Ethnic Groups of Daily Smokers

**DOI:** 10.3390/healthcare5010002

**Published:** 2017-01-11

**Authors:** Julia N. Soulakova, Ryan R. Danczak

**Affiliations:** 1 Burnett School of Biomedical Sciences, College of Medicine, University of Central Florida, 6900 Lake Nona Blvd, Orlando, FL 32827, USA; 2 Department of Statistics, University of Nebraska-Lincoln, 340 Hardin Hall-North, Lincoln, NE 68583, USA; ryandanczak21@gmail.com

**Keywords:** cigarette flavoring, every day smoker, national survey, population-based study, smoking behaviors, smoking status

## Abstract

*Introduction*: The aims of this study were to evaluate whether menthol smoking and race/ethnicity are associated with nicotine dependence in daily smokers. *Methods*: The study used two subsamples of U.S. daily smokers who responded to the 2010–2011 Tobacco Use Supplement to the Current Population Survey. The larger subsample consisted of 18,849 non-Hispanic White (NHW), non-Hispanic Black (NHB), and Hispanic (HISP) smokers. The smaller subsample consisted of 1112 non-Hispanic American Indian/Alaska Native (AIAN), non-Hispanic Asian (ASIAN), non-Hispanic Hawaiian/Pacific Islander (HPI), and non-Hispanic Multiracial (MULT) smokers. *Results*: For larger (smaller) groups the rates were 45% (33%) for heavy smoking (16+ cig/day), 59% (51%) for smoking within 30 min of awakening (Sw30), and 14% (14%) for night-smoking. Overall, the highest prevalence of menthol smoking corresponded to NHB and HPI (≥65%), followed by MULT and HISP (31%–37%), and then by AIAN, NHW, and ASIAN (22%–27%) smokers. For larger racial/ethnic groups, menthol smoking was negatively associated with heavy smoking, not associated with Sw30, and positively associated with night-smoking. For smaller groups, menthol smoking was not associated with any measure, but the rates of heavy smoking, Sw30, and night-smoking varied across the groups. *Conclusions*: The diverse associations between menthol smoking and nicotine dependence maybe due to distinction among the nicotine dependence measures, i.e., individually, each measure assesses a specific smoking behavior. Menthol smoking may be associated with promoting smoking behaviors.

## 1. Introduction

Cigarette smoking has been shown to be associated with a number of life-threatening diseases, e.g., cardiovascular and obstructive lung diseases, early deaths, productivity loss, and financial trouble to society, as well as a substantial burden on the healthcare system [1]. Menthol cigarette smoking has received particular attention in the nicotine and tobacco research literature. Menthol cigarette smoking may lead to significantly larger puff volumes, higher cotinine intake, and may increase the risk of a heart attack [2,3,4]. Menthol smoking may also contribute to reinforcing smoking behavior [5,6,7], increased difficulty abstaining from smoking in places where smoking is banned [8], and may delay quit attempts and smoking cessation [9,10,11].

A few studies have found evidence that menthol smoking leads to increased levels of nicotine dependence [12,13], e.g., menthol cigarette smoking was related to a shorter time to the first cigarette after waking up in White and Black females [4] as well, as Black daily smokers who smoked five or more cigarettes per day (cig/day) [14], smoking within 5 min after waking up in daily smokers who smoked 6–10 cig/day [15], and night-smoking, i.e., waking up at night to smoke, in patients seeking smoking cessation treatment [16]. However, a comprehensive review of publications addressing impact of menthol smoking on nicotine dependence (published or available on-line prior to 2014) has revealed that menthol smoking is not associated with greater number of cig/day, based on 29 publications, smoking within 30 min from waking up (Sw30), based on nine publications, and several other validated and widely-used nicotine dependence measures [17]. However, two studies have showed that menthol smoking is positively associated with night-smoking [10,16], and no other studies have explored this relationship [17].

Impact of menthol smoking on nicotine dependence may be different for diverse racial/ethnic groups, e.g., among Black smokers, menthol smokers had shorter mean time to the first cigarette after waking up, but among White smokers the difference was not significant [14]. However, these possible racial/ethnic differences in the impact of menthol smoking on nicotine dependence have not been adequately addressed.

A number of studies discussed the overall effect of race/ethnicity on nicotine dependence, e.g., [4,14,15], and prevalence of menthol smoking. In particular, several studies showed that NHB smokers have the highest rate of menthol smoking among several other racial/ethnic groups [3,18,19,20]. In addition, one study estimated the prevalence of menthol smoking among 25–64 year old daily smokers, using 2005 National Health Interview Survey data [18]. The prevalence of menthol cigarette smokers among male (female) smokers was about 70% (78%) for NHB, 17% (36%) for Hispanic (HISP), and 15% (25%) for non-Hispanic White (NHW) smokers [18]. Another study detected the high rates of menthol smoking among younger Non-Hispanic Asian (ASIAN) and Non-Hispanic American Indian/Alaska Native (AIAN) smokers, e.g., the prevalence of menthol smoking among ASIAN smokers was about 52% for 12–17 year olds, 36% for 18–25 year olds and 29% for 26+ year olds, and among AIAN smokers the rates were about 35% for 12–17 year olds, 27% for 18–25 year olds and 23% for 26+ year olds [21]. Another related study examined the nicotine dependence and menthol smoking in Native Hawaiian and Filipino daily smokers who had high risks for tobacco-caused lung cancer [15]. The observed rates of menthol smoking were 87% for Native Hawaiians, 72% for Filipinos, and 47% for Whites [15].

It has been also mentioned that tobacco industries have mainly targeted NHB [6,22,23]. This could contribute to the high rate of menthol smoking among this subpopulation [3,18,19,20]. In addition, the rates of lung cancer and other smoking-related adverse health outcomes are disproportionate among diverse racial/ethnic groups [24,25], which may be due to racial/ethnic differences in the patterns of smoking behaviors, e.g., nicotine dependence and menthol smoking.

Despite the importance of prior studies addressing menthol smoking, nicotine dependence, and racial/ethnic disparities in smoking behaviors, results of these studies cannot be used to assess whether racial/ethnic disparities in nicotine dependence are contingent on menthol smoking status. Moreover, the majority of prior studies were not comprehensive in terms of racial/ethnic representation. To help close this gap, we used self-reports of smoking behaviors for seven (most represented in the US) racial/ethnic groups, including NHW, NHB, HISP, AIAN, ASIAN, non-Hispanic Hawaiian/Pacific Islander (HPI), and non-Hispanic Multiracial (MULT). The specific goals of this study were to (1) estimate the overall rates of menthol smoking for each racial/ethnic group of daily smokers and determine if the rates differ significantly across racial/ethnic groups; (2) explore and determine significance of associations between menthol smoking and nicotine dependence within each racial/ethnic group of daily smokers; and (3) assess possible differences in the odds of being more nicotine dependent associated with menthol smoking and race/ethnicity, while adjusting for other important factors.

## 2. Material and Methods

### 2.1. Dataset and Primary Measures

The study used the 2010–2011 Tobacco Use Supplement to the Current Population Survey data that are publicly available [26]. The Supplement has been implemented as a part of the Survey by the U.S. Census Bureau since 1992. It is sponsored by the National Cancer Institute and Centers for Disease Control and Prevention. About 240,000 households are surveyed each period [26]. The Supplement data are commonly used to estimate the smoking prevalence and assess other tobacco-related information. Our study used two subsamples of daily smokers who reported their sociodemographic and smoking-related information. The subsample for larger racial/ethnic groups consisted of 81.6% (16,071) NHW, 10.7% (1700) NHB, and 7.7% (1078) HISP daily smokers. The subsample for smaller racial/ethnic groups consisted of 19.1% (310) AIAN, 40.9% (345) ASIAN, 4.1% (53) HPI, and 35.9% (404) MULT daily smokers. Sociodemographic characteristics of daily smokers for each subsample are depicted in Table 1.

The study considered three (binary) nicotine dependence measures, i.e., heavy smoking status (smoking 1–15 cig/day, smoking 16+ cig/day), Sw30 (yes, no), and night-smoking (yes, no). The heavy smoking status and night-smoking were defined using responses to the survey questions “On average, about how many cigarettes do you now smoke each day?” and “Do you sometimes awaken during the night to have a cigarette?” respectively. The Sw30 measure was defined as follows. First, all daily smokers were asked “How soon after you wake up do you typically smoke your first cigarette?” If the respondent could not specify the exact time, then the respondent was asked the follow-up question “Would you say you smoke your first cigarette of the day within the first 30 min?” Responses to these two questions were pooled to define (approximately) the Sw30 measure.

The primary independent measure was menthol smoking status (menthol, non-menthol), which was defined using responses to the survey question “Do you usually smoke menthol or non-menthol cigarettes?” Sample counts for this and additional factors are listed in Table 1.

### 2.2. Statistical Methods

The larger and smaller subsamples were analyzed separately. Rao-Scott chi-square tests were used when testing for associations between the measures [27,28]. Significance level was fixed at the 5% level for each global test. In the case of a significant result for larger (smaller) racial/ethnic groups, two post-hoc comparisons between NHB and NHW, and HISP and NHW (AIAN and MULT, and ASIAN and MULT) were performed.

To assess goal three, we used multiple logistic regressions for complex survey data. For each nicotine dependence measure, the model assessed possible effects of covariates on the odds of having higher level of nicotine dependence, i.e., the reference levels were smoking 1–15 cig/day (on average), not smoking within 30 min from awakening and not smoking at night. For the larger racial/ethnic groups, the initial models included two-way interactions between race/ethnicity and all other characteristics. For smaller racial/ethnic groups (the HPI daily smokers were not included in the models due to the small group sample size), the observed percentages were included in the tables. The initial models included the interaction between race/ethnicity and menthol smoking. If an interaction was not significant at the 5% level and was the least significant among remaining in the model interactions, it was deleted and the model was refitted, until only significant interactions remained or all interactions were excluded. As a result, the final models for heavy smoking contained the significant interactions between race/ethnicity and menthol smoking (*p* = 0.001), and age (*p* = 0.001); the final model for Sw30—between race/ethnicity and age (*p* = 0.044), gender (*p* = 0.001) and region (*p* < 0.001), and the final model for night-smoking—between race/ethnicity and menthol smoking (*p* = 0.019), and education (*p* < 0.001). The final models for smaller racial/ethnic groups contained only the main effects.

Bonferroni adjustments were used for the post hoc comparisons, e.g., model-based pair-wise comparisons between the racial/ethnic groups were performed within each group of menthol and non-menthol smokers; in these cases, the adjusted *p*-value (ap) and simultaneous confidence interval (CI) are reported; for simplicity, 99.99% CI denotes 99.9875% CI. All analyses adjusted for the Survey’s design specifics, i.e., 160 replicate weights and the main weight were downloaded, merged with the Supplement data, and used in the analyses [26]. Computing was done using the survey package in SAS/STAT^®^ 9.4 (SAS Institute Inc., Cary, NC, USA) [29].

## 3. Results

### 3.1. Goal 1: Racial/Ethnic Differences in the Overall Rates of Menthol Smoking

Overall, NHB and HPI daily smokers had the highest prevalence of menthol smoking (both rates are about or above 65%), followed by MULT and HISP (31%–37%), and AIAN, NHW, and ASIAN daily smokers had the lowest prevalence of menthol smoking (22%–27%), see Table 2. The rates were significantly different across the larger (*p* < 0.001) and smaller (*p* < 0.001) racial/ethnic groups. Rates of menthol smoking were significantly higher for NHB (OR = 12.2; CI = 10.6:14.0; *p* < 0.001) and HISP (OR = 1.4; CI = 1.2:1.7; *p* < 0.001) than for NHW. The rates were also significantly higher for HPI (OR = 3.2; 98.3% CI = 1.3:7.9; ap = 0.003) than for MULT, but lower for ASIAN (OR = 0.5; 98.3% CI = 0.3:0.7; ap < 0.001) than for MULT. The difference between the rates for AIAN and MULT was not significant after the multiplicity adjustments (OR = 0.6, 98.3% CI = 0.4:1.1).

### 3.2. Goal 2: Associations between Menthol Smoking and Nicotine Dependence within Each Racial/Ethnic Group of Daily Smokers

#### 3.2.1. Differences in Prevalence of Menthol Smoking Associated with Nicotine Dependence

Within each racial/ethnic group (see Table 2) the proportion of menthol smokers was consistently lower among heavy smokers (i.e., those who smoked 16+ cig/day) than among less heavy smokers (i.e., those who smoked 1–15 cig/day). The proportions of menthol heavy smokers (in the order from smallest to largest) corresponded to ASIAN, NHW, AIAN, HISP, MULT, HPI, and NHB smokers. There was no clear pattern observed for Sw30 and night-smoking: the proportion of menthol smokers with the higher level of nicotine dependence could be larger or smaller than the proportion of menthol smokers with the lower level of nicotine dependence, depending on the measure and race/ethnicity. Table 2 also illustrates that the highest proportion of menthol smokers for each level of each nicotine dependence measure corresponded to NHB smokers (not counting the proportion of HPI smokers who wake up at night to smoke due to the insufficient sample size).

#### 3.2.2. Differences in Nicotine Dependence Associated with Menthol Smoking

The overall rates of heavy smoking, Sw30 and night-smoking, were 44.8%, 58.9%, and 14.0%, respectively. This prevalence pattern indicates that Sw30 was the most prevalent behavior, followed by heavy smoking while night-smoking was the least prevalent behavior. This pattern was also observed for larger and smaller racial/ethnic groups: for larger (smaller) groups the rate of heavy smoking was 45.4% (32.9%), the rate of Sw30 was 59.3% (51.2%) and the rate of night-smoking was 14.0% (14.4%).

Table 3 depicts the rates of higher levels of nicotine dependence for each measure separately for menthol and non-menthol cigarette smokers within each racial/ethnic group. For example, the Table illustrates that among NHW menthol smokers, there are about 44% of heavy smokers and 56% of non-heavy smokers. We note that because the proportions of HPI menthol and non-menthol smokers who reported smoking at night were very low, the HPI smokers were excluded from all comparisons concerning night-smoking.

For larger racial/ethnic groups of menthol smokers, all nicotine dependence measures were significantly associated (all *p* < 0.001) with race/ethnicity. Specifically, NHB (OR = 0.4; CI = 0.3:0.4; *p* < 0.001) and HISP (OR = 0.3; CI = 0.2:0.4; *p* < 0.001) had significantly lower rates of heavy smoking, and HISP had lower rate of Sw30 (OR = 0.6; CI = 0.5:0.7; *p* < 0.001) when compared to the NHW smokers. However, the NHB smokers had higher rate of night-smoking than NHW (OR = 1.8; CI = 1.5:2.2; *p* < 0.001).

For smaller racial/ethnic groups of menthol smokers, the only significant association with race/ethnicity was observed for heavy smoking (*p* = 0.032) but the specific comparisons relative to MULT smokers did not reveal any significant differences (after multiplicity adjustments).

For larger racial/ethnic groups of non-menthol smokers, heavy smoking and Sw30 were significantly associated (both *p* < 0.001) with race/ethnicity. In particular, NHB (OR = 0.6; CI = 0.4:0.7; *p* < 0.001) and HISP (OR = 0.3; CI = 0.2:0.4; *p* < 0.001) had significantly lower rates of heavy smoking when compared to the NHW smokers. Additionally, HISP (OR = 0.4; CI = 0.3:0.5; *p* < 0.001) had the lower rate of Sw30 when compared to the NHW smokers. Night-smoking was not significantly associated with race/ethnicity among non-menthol smokers.

For smaller racial/ethnic groups of non-menthol smokers, all nicotine dependence measures were significantly associated with race/ethnicity (all *p*’s < 0.007). ASIAN non-menthol smokers had lower rates of heavy smoking (OR = 0.4; 98.3% CI = 0.2:0.7; *p* < 0.001), Sw30 (OR = 0.6; 98.3% CI = 0.4:0.9; *p* = 0.002), and night-smoking (OR = 0.3; 98.3% CI = 0.2:0.7; *p* < 0.001) when compared to the MULT smokers. The other differences were not significant.

### 3.3. Goal 3: Differences in the Odds of Being More Nicotine Dependent Associated with Menthol Smoking and Race/Ethnicity while Adjusting for Other Important Factors

Table 4 presents the final regression model for each nicotine dependence measure. Effect of menthol smoking on heavy smoking differed across larger racial/ethnic groups. For menthol smokers, the odds of heavy smoking were significantly lower for NHB and HISP than they were for NHW smokers (NHB: OR = 0.2, 99.99% CI = 0.2:0.3; HISP: OR = 0.3, 99.99% CI = 0.2:0.5; both ap’s < 0.001). A similar pattern was observed for non-menthol smokers (NHB: OR = 0.4, 99.99% CI = 0.3:0.6; HISP: OR = 0.3, 99.99% CI = 0.2:0.4; both ap’s < 0.001) when compared to the NHW smokers. We note that the significant interaction effect is due to the different patterns for NHB an HISP smokers: among menthol smokers NHB smokers had lower odds of heavy smoking than HISP smokers (OR = 0.7) while among non-menthol smokers, NHB smokers had higher odds of heavy smoking than HISP smokers (OR = 1.3), but significance of this comparison was not assessed.

Effect of menthol smoking on Sw30 did not differ across larger racial/ethnic groups. The overall effect of menthol smoking was significant only at 10% level with OR = 0.9 (CI = 0.8:1.0; *p* = 0.093) for menthol versus non-menthol smoking. The overall racial/ethnic effect was significant but comparisons to the NHW group resulted in the only significant difference, i.e., HISP daily smokers had lower odds of Sw30 than NHW daily smokers (OR = 0.5; CI = 0.4:0.6; *p* < 0.001).

Effect of menthol smoking on night-smoking differed significantly across the racial/ethnic groups. Among menthol smokers, NHB smokers had higher odds while HISP smokers had lower odds of night-smoking when compared to the NHW smokers (NHB: OR = 1.9, 99.99% CI = 1.1:3.1, ap = 0.012; HISP: OR = 0.0, 99.99% CI = 0.0:0.6; ap = 0.011). Among non-menthol smokers, the only significant difference was in the odds for HISP and NHW smokers (OR = 0.0, 99.99% CI = 0.0:0.3; ap = 0.002).

For smaller racial/ethnic groups, menthol smoking was not significantly associated with any of the nicotine dependence measures but race/ethnicity was significantly associated with each nicotine dependence measure. ASIAN smokers had lower odds than MULT smokers with respect to each measure (for heavy smoking: OR = 0.4, CI = 0.3:0.6, *p* < 0.001; for Sw30: OR = 0.6, CI = 0.4:0.9, *p* = 0.010; and for night-smoking: OR = 0.6, CI = 0.4:0.9, *p* = 0.015).

There was an additional finding that survey mode is significantly associated with the odds of being more nicotine dependent. The ORs for phone versus in-person interviews ranged from 0.8 to 0.9 for larger racial/ethnic groups and were about 0.6 for smaller racial/ethnic groups indicating that the phone interviews are associated with the smaller odds of being more nicotine dependent than are the in-person interviews.

## 4. Discussion

The 2010–2011 Supplement data analyses indicated that the prevalence of menthol smoking among daily smokers remains disproportionate across racial/ethnic groups in the US. The highest rates of menthol smoking (in the order from the highest rate to the lowest rate) were observed for NHB, HPI, MULT, HISP, AIAN, NHW, and ASIAN (significance of the exact order has not been explored). The disproportional rates of menthol smoking across the racial/ethnic groups could be due to cultural differences, as well as several other related factors. For example, menthol cigarettes have been more widely advertised among the racial/ethnic minorities and neighborhoods with lower socio-economic status, potentially contributing to the increased rates of menthol smoking for these subpopulations [30,31,32,33,34]. Moreover, there are racial/ethnic differences in the perception of harm of menthol smoking, i.e., menthol cigarettes have been wrongly viewed as a healthier alternative to the regular cigarettes, also possibly contributing to the higher prevalence of menthol smoking [35].

The study also indicates that race/ethnicity and menthol smoking may jointly effect nicotine dependence, i.e., diverse racial/ethnic groups of daily smokers may have higher or lower odds of being more nicotine dependent, contingently on their menthol smoking status. Specifically, among NHW, NHB, and HISP menthol smokers, the highest predicted odds of heavy smoking (in the order from highest to smallest) were observed for NHW, HISP, and NHB daily smokers, while among non-menthol smokers the higher odds corresponded to NHB than HISP smokers; all comparisons to NHW smokers were significant. Additionally, among NHW, NHB, and HISP menthol smokers, the highest predicted odds of night-smoking (in the order from highest to smallest) were observed for NHB, NHW, and HISP daily smokers (both comparisons to NHW group were significant), but among non-menthol smokers, the rates were similar for NHB and NHW, and significantly different for NHW and HISP. A similar investigation with respect to smaller racial/ethnic groups did not indicate such a joint effect, possibly due to low statistical power.

Analyses, not controlling for the other covariates, indicated that the highest rates of Sw30 (all rates are above 60%) correspond to NHW, NHB, and AIAN non-menthol smokers and AIAN non-menthol smokers. However, after adjusting for the other covariates, significance of the joint effect between race/ethnicity and menthol smoking status was not confirmed.

The additional results that menthol smoking (overall) is negatively associated with heavy smoking, positively associated with night-smoking, and not associated with Sw30, are consistent with the ones reported in prior studies [4,5,10,12,13,14,15,16,17]. This discrepancy may be due to dissimilarities among the measures. First, the prevalence of being more nicotine dependent was rather distinct depending on a measure: while the overall rate of Sw30 was about 59%, the rate of night-smoking was only 14%. Second, the Pearson correlation between time to the first cigarette of the day and the number of cig/day (when both measures are continuous, n = 19,406), had a value of –0.34 (*p* < 0.001) indicating a rather mild (negative) linear relationship between these measures, i.e., daily smokers who smoked more cig/day tended to have shorter time to the first cigarette of the day, but the linear relationship was not strong. Third, a prior study of long-term smoking cessation in patients seeking smoking cessation treatment had a relevant conclusion: among these nicotine dependence measures, night-smoking was the only predictor of long-term smoking cessation [16]. Thus, different effect of menthol smoking on these smoking behaviors could be, at least in part, due to the distinction among the nicotine dependence measures.

The study has several limitations. First, the study revealed that survey mode significantly influenced daily smoker’s reports of smoking behaviors: phone interviews were associated with lower odds of being more nicotine dependent than in-person interviews. Reporting more favorable information could be due to social desirability or another type of the response bias. A similar finding has been observed with respect to current smoking and several other smoking-related behaviors [36,37,38]. Despite the significant effect of the survey mode, we do not anticipate that the study findings are affected disproportionally across the racial/ethnic groups. Second, there was an insufficient sample size for some racial/ethnic groups which limited our statistical analyses, e.g., the sample size for the HPI smokers prohibited detailed investigations and comparisons with the other racial/ethnic groups of smokers. Additionally, two considered subsamples for larger and smaller racial/ethnic groups have very mixed compositions, which may limit the findings. Third, the study explored only a limited number of factors and the observed results could be due to confounding effects not considered in the study.

We also note that validation of the considered measures for nicotine dependence is out of scope of this study. While the mean number of cig/day and time to the first cigarette of the day are the drawing factors for the Fagerström Test for Nicotine Dependence (FTND) composite scores [17], having higher levels on an individual measure does not necessarily correspond to the higher level of nicotine dependence according to the FTND and/or meeting criteria for tobacco use disorder, e.g., see [17]. We used these nicotine dependence measures because these measures have been previously addressed in the behavioral literature as features associated with tobacco use disorder [17].

## 5. Conclusions

Positive association of menthol smoking with night-smoking may suggest that menthol cigarettes should not be viewed as a healthier alternative to regular cigarettes and menthol may be more than simply a flavoring additive because it may endorse smoking behaviors. Negative association of menthol smoking with heavy smoking and no association with Sw30 do not necessarily point to a contradiction. While all considered nicotine dependence measures are related and commonly used as markers of nicotine dependence, each one describes a particular smoking behavior and, thus, when considered individually, represents a distinct type and/or severity of addiction.

The considered nicotine dependence behaviors had unique prevalence patterns. The most prevalent behavior was Sw30: NHW, NHB, AIAN, and MULT smokers had the highest rates (57%–83%), followed by HISP and ASIAN smokers (about 41%), and HPI smokers (30%). The second most prevalent behavior was heavy smoking: the highest rates were observed for NHW (50%), followed by AIAN and MULT (40%–44%), and NHB, HISP, ASIAN, and HPI smokers (22%–25%). The least prevalent behavior was night-smoking: the highest rates corresponded to NHB, AIAN, and MULT (18%–20%), followed by NHW, HISP, and ASIAN (10%–13%), and HPI smokers (about 2%).

Future investigation of the effect of menthol smoking on nicotine dependence using other nicotine dependence measures, e.g., FTND, and samples better representing racial/ethnic minorities are of great importance. Studies dealing with a lack of FTND information could consider investigating available nicotine dependence measures simultaneously, e.g., the lowest addiction level could correspond to daily smokers who are light smokers, do not smoke within 30 min of awakening and do not engage in night-smoking, while the highest addiction level could correspond to daily smokers who are heavy smokers, smoke within 30 min from awakening, and engage in night-smoking.

## Figures and Tables

**Table 1:** Sample characteristics of the daily smokers for larger and smaller racial/ethnic groups.

Characteristic	NHW, NHB and HISP Smokers		AIAN, ASIAN, HPI and MULT Smokers	
	Group Count	Population Count Percent (%)	Group Count	Population Count Percent (%)
**Age**				
18–24	1512	12.7	99	12.5
25–44	7185	38.1	504	47.6
45–64	8253	40.9	418	33.1
65+	1890	8.2	91	6.8
**Gender**				
Male	9087	52.5	597	59.4
Female	9762	47.5	515	40.6
**Highest Level of Education**				
Less than High school	3154	18.1	178	13.5
High school (or equivalent)	7957	41.5	414	36.4
Some college or a Bachelor’s degree	7286	38.2	484	46.1
Graduate degree	452	2.2	36	4.1
**Region**				
Northeast	3654	16.8	121	12.2
Midwest	5408	27.4	259	19.5
South	6551	40.7	244	29.6
West	3236	15.1	488	38.6
**Metropolitan Status**				
Metropolitan	13,664	78.8	774	82.5
Non-metropolitan	5185	21.2	338	17.5
**Survey Mode**				
Phone	11,041	56.2	595	50.4
In-person	7808	45.8	517	49.6
**Menthol Smoking Status**				
Menthol	5256	30.4	344	29.8
Non-menthol	13,593	69.6	768	70.2
**Subsample and Population Count**	18,849	24,918,980	1112	1,232,271

**Table 2:** Proportions (%, with 95% confidence intervals) of menthol smokers for each level of nicotine dependence and race/ethnicity.

Nicotine Dependence Measure	NHW	NHB	HISP	AIAN	ASIAN	HPI	MULT
Smoking 16+ cigarettes per day	21.3 20.0:22.2	69.2 64.0:74.4	27.1 20.4:33.7	24.9 15.3:34.4	20.8 11.0:30.6	61.2 20.7:100.0	33.2 24.6:41.8
Smoking 1–15 cigarettes per day	26.6 25.3:27.8	82.6 80.1:85.1	32.4 28.6:36.3	29.3 19.9:38.6	21.9 15.3:28.6	65.8 48.6:83.1	39.2 31.7:46.6
Smoking within 30 min	22.6 21.5:23.7	78.4 75.4:81.4	34.2 28.9:39.6	26.4 18.1:34.7	20.7 13.6:27.7	72.3 41.2:100.0	37.1 29.5:44.7
Not smoking within 30 min	25.8 24.4:27.2	80.4 77.0:83.8	29.1 24.9:33.3	28.9 17.9:39.9	22.4 14.4:30.4	61.6 42.7:80.4	36.3 26.8:45.7
Night-smoking	23.5 21.3:25.6	84.1 79.6:88.6	38.2 29.1:47.4	34.5 15.2:53.7	35.1 19.5:50.7	100.0 *	30.1 17.3:42.8
Not smoking at night	23.9 2.0:24.8	78.0 75.5:80.4	30.2 26.6:33.8	25.6 18.3:32.9	20.2 14.1:26.4	63.9 48.1:79.8	38.3 31.7:44.9
Overall	23.8 23.0:24.7	79.2 77.0:81.5	31.2 27.7:34.6	27.3 20.6:34.0	21.7 16.1:27.2	64.8 49.2:80.5	36.8 30.8:42.7

* The group sample size is 5.

**Table 3:** Percentage of daily smokers with higher levels of nicotine dependence for each nicotine dependence measure, race/ethnicity and menthol/non-menthol smoking status.

Nicotine Dependence Measure	NHW	NHB	HISP	AIAN	ASIAN	HPI	MULT
Menthol smokers							
Heavy smokers	44.3	22.1	20.2	39.8	21.4	20.8	36.4
Smoke within 30 min of waking up	57.8	58.8	44.6	60.5	40.5	33.5	57.9
Night-smokers	13.1	21.3	15.3	24.8	15.6	3.8	15.0
Non-menthol smokers							
Heavy smokers	51.8	37.5	24.6	45.2	22.5	24.3	42.5
Smoke within 30 min of waking up	62.0	61.7	38.8	63.5	42.9	23.7	57.0
Night-smokers	13.4	15.4	11.2	17.7	8.0	0.0	20.3
Overall							
Heavy smokers	50.0	25.3	23.2	43.7	22.3	22.1	40.3
Smoke within 30 mins of waking up	61.0	59.4	40.6	62.7	42.4	30.1	57.3
Night-smokers	13.3	20.1	12.5	19.7	9.6	2.4	18.3

**Table 4:** Results of multiple logistic regressions: main effects and *p*-values.

Main Effect *	NHW, NHB and HISP Daily Smokers			AIAN, ASIAN and MULT Daily Smokers		
	Heavy Smoking	Sw30	Night-Smoking	Heavy Smoking	Sw30	Night-Smoking
Menthol smoking	S	NS	0.001	NS	NS	NS
Race/ethnicity	S	S	S	S	0.023	0.040
Age	S	S	S	0.016	NS	NS
Gender	S	NS	NS	0.049	NS	NS
Education	S	S	S	NS	0.001	NS
Region	S	S	NS	NS	NS	NS
Metropolitan status	S	S	NS	NS	NS	NS
Survey mode	0.014	0.001	S	0.012	0.003	0.034
Model fit: Wald chi-square statistic (degrees of freedom) and *p*-value	1843.5 (23) S	635.4 (29) S	255.3 (23) S	72.2 (15) S	61.3 (15) S	25.8 (15) 0.040

* NS denotes non-significant (at 5% level) effects; S denotes *p* < 0.001.
